# Supplementary material for: Targeted deletion of the RNA-binding protein Caprin1 leads to progressive hearing loss and impairs recovery from noise exposure in mice
Source: Sci Rep. 2022 Feb 14;12:2444. doi: 10.1038/s41598-022-05657-2 (PMC8844073; doi:10.1038/s41598-022-05657-2)
Supplement: Supplementary file 1 — Supplementary Methods. [file 41598_2022_5657_MOESM1_ESM.docx]

**Supplementary Methods**

Antibodies.

*Vibratome sections.* Primary antibodies: rabbit anti-Caprin1, raised against amino acids 356-709, a region encoded by exons 10-19 was used at1:200 (Proteintech Europe, #15112-1-AP); mouse (­­­­IgG­_2a_) anti-TuJ1, 1:500 (Covance, #MMS-435P). Secondary antibodies: goat anti-(rabbit IgG) conjugated to Alexa Fluor 633, 1:500 (#A-21070); goat anti-(mouse IgG_2a_) conjugated to Alexa Fluor 488, 1:500 (#A-21131) – both Invitrogen.

*Cochlear whole mounts.* Primary antibodies: mouse (IgG_1_) anti-CtBP2, 1:400 (BD Biosciences, #612044); mouse (IgG_2a_) anti-GluA2, 1:200 (Millipore, #MAB397); rabbit anti-Myosin7a, 1:200 (Proteus Biosciences, #25-6790). Secondary antibodies: goat anti-(mouse IgG_1_) conjugated to Alexa Fluor 568, 1:500 (#A-21124); goat anti-(mouse IgG_2a_) conjugated to Alexa Fluor 488, 1:500 (#A-21131); goat anti-(rabbit IgG) conjugated to Alexa Fluor 405, 1:300 (#A-31556) – all Invitrogen.

*Ex-vivo cochlear cultures.* Primary antibodies: rabbit anti-Caprin1, 1:500 (Proteintech Europe, #15112-1-AP); goat anti-TIA-1 (C-20), 1:300 (SantaCruz Biotechnology, #sc-1751); mouse (IgG_1_) anti-HuR (3A2), 1:500 (SantaCruz Biotechnology, #sc-5261). Secondary antibodies: donkey anti-(rabbit IgG) conjugated to Alexa Fluor 647, 1:100 (#A-31573, Thermofisher); donkey anti-(goat IgG) conjugated to Alexa Fluor 488, 1:1000 (#A-11055, Thermofisher).
