# Supplementary material for: Targeted deletion of the RNA-binding protein Caprin1 leads to progressive hearing loss and impairs recovery from noise exposure in mice
Source: Sci Rep. 2022 Feb 14;12:2444. doi: 10.1038/s41598-022-05657-2 (PMC8844073; doi:10.1038/s41598-022-05657-2)
Supplement: Supplementary file 2 — Supplementary Figure 1. [file 41598_2022_5657_MOESM2_ESM.pdf]

Targeted deletion of the RNA-binding protein *Caprin1* leads to progressive hearing loss and impairs recovery from noise exposure in mice

Lisa S. Nolan[1,2], Jing Chen[2], Ana-Claudia Goncalves[1], Anwen Bullen[1], Emily R. Towers[1], Karen P. Steel[2], Sally J. Dawson[1*] and Jonathan E. Gale[1*]

[1]UCL Ear Institute, 332 Gray's Inn Road, London. WC1X 8EE

[2]Wolfson Centre for Age-Related Diseases, King's College London, Guy's Campus, London. SE1 1UL.

SUPPLEMENTARY FIGURE 1

SUPPLEMENTARY FIGURE 1

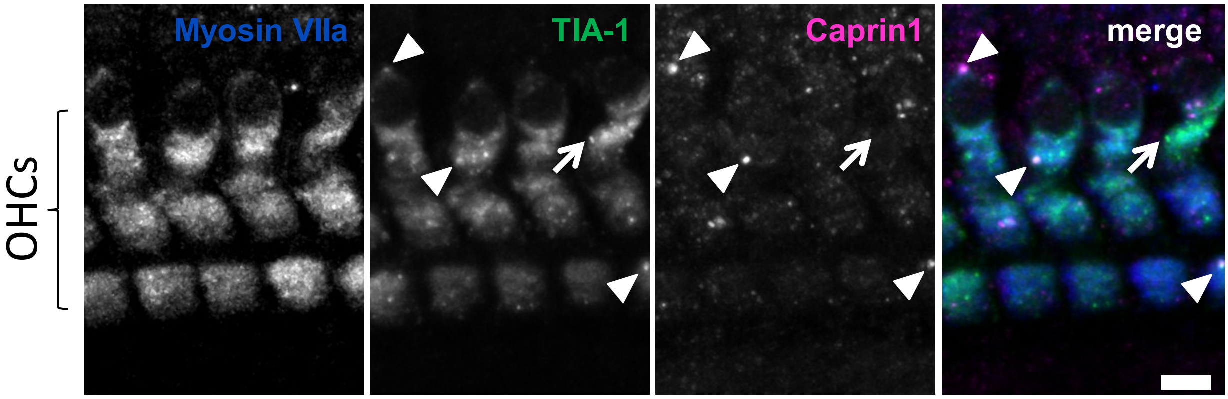

Supplementary Figure 1. Noise exposure induced stress granule formation in outer hair cells *in vivo*. P18-20 C57/BL6 mice subjected to noise (100dbSPL, 2 hours). Vibratome cochlear slices (from cochlea fixed at the end of the noise exposure) were immunolabelled with myosinVIIa (blue), TIA-1 (green), Caprin1 (red). Stress granules were identified in the OHC region of the organ of Corti. Arrowheads indicate stress granules positive for both TIA-1, and Caprin1 or in some cases Caprin1-negative stress granules (arrow). Scale bar, 5 µm.
